# Supplementary material for: Simulation design to find the welfare impacts of livestock trading and disease transmission
Source: PLoS One. 2024 Nov 20;19(11):e0310213. doi: 10.1371/journal.pone.0310213 (PMC11578522; doi:10.1371/journal.pone.0310213)
Supplement: S1 Appendix — (PDF) [file pone.0310213.s001.pdf]

Appendix

Proof of Proposition 1

Proof. First, let us check if there are transactions where $(1-\lambda, 1-\delta)$ group and $(\lambda, 1-\delta)$ group trade. In order for transactions between the two groups to occur, the condition that the one group becomes the consumer and the other group becomes the supplier is required (the ranges of λ overlap). However, the possibility is negated by the following relationship.:

$$\text{Demand} < \frac{\delta(L^b - L^s)}{\eta(\mu_D + \omega)[\delta L^b + (1-\delta)L^s]} < \frac{\eta(\mu_D + \omega)L^s + \delta(L^b - L^s)}{\eta(\mu_D + \omega)[\delta L^b + (1-\delta)L^s]} \leq \text{Supply}.$$

Second, households included in the $(\lambda, 1-\delta)$ category eventually become 'Always Buyer' because the following relationship is contradicted in relation to the households included in the (λ, δ) category.

$$\frac{L^b}{\delta L^b + (1-\delta)L^s} \leq \lambda < \frac{L^s}{\delta L^b + (1-\delta)L^s},$$
$$\text{a contradiction} \because L^b > L^s \text{ and } \delta L^b + (1-\delta)L^s > 0.$$

Thus, livestock transactions between the (λ, δ) group and the $(1-\lambda, 1-\delta)$ group are only possible. ■

Proof of Proposition 2

Proof. Since households in the $(1-\lambda, \delta)$ group are the always seller and households in the $(\lambda, 1-\delta)$ group are the always buyer, only if households in the (λ, δ) group become sellers, there is a possibility of spreading infected animals through livestock trading. From Proposition 1 if the (λ, δ) group sells livestock ($X_{i1}^{\lambda,\delta} < 0$) then $X_{i1}^{1-\lambda,1-\delta} > 0$ if

$$\begin{aligned}
\frac{L^b}{\delta L^b + (1-\delta)L^s} &< \frac{\delta(L^b - L^s)}{\eta(\mu_D + \omega)[\delta L^b + (1-\delta)L^s]} \\
L^b \eta(\mu_D + \omega) &< \delta(L^b - L^s) \\
\frac{L^s}{L^b} &< 1 - \frac{\eta}{\delta}(\mu_D + \omega) \qquad \text{if } \eta(\mu_D + \omega) < \delta.
\end{aligned}$$

Therefore, if $0 < \eta(\mu_D + \omega) < \delta$ and $\frac{L^s}{L^b} < 1 - \frac{\eta}{\delta}(\mu_D + \omega)$, the households with the initial infectious livestock can make a deal in the sick animals. ■

Proof of Proposition 3

Proof. Suppose $\sum_{m=1}^{M-2}(L^{M-1} - L^m)\delta^m - \delta^M L^M > 0$. If $\sum_{m=1}^{M-2}(L^{M-1} - L^m)\delta^m - \delta^M L^M - L^{M-1} > 0$, then $\lambda\eta(\mu_D + \omega) > \dfrac{\sum_{m=1}^{M-2}(L^{M-1} - L^m)\delta^m - \delta^M L^M}{\sum_{m=1}^{M-2}(L^{M-1} - L^m)\delta^m - \delta^M L^M - L^{M-1}} > 1$. But this contradicts $0 < \lambda\eta(\mu_D + \omega) < 1$. Now if $\sum_{m=1}^{M-2}(L^{M-1} - L^m)\delta^m - \delta^M L^M - L^{M-1} < 0$, similarly the relationship $0 < \lambda\eta(\mu_D + \omega) < \dfrac{\sum_{m=1}^{M-2}(L^M - L^m)\delta^m - \delta^M L^M}{\sum_{m=1}^{M-2}(L^{M-1} - L^m)\delta^m - \delta^M L^M - L^{M-1}} < 0$, a contradiction from the fact that $\lambda, \eta, \mu_D, \omega \in (0, 1]$. Therefore, it can proved that $\sum_{m=1}^{M-2}(L^{M-1} - L^m)\delta^m - \delta^M L^M < 0$ is a true statement. Thus,

$$
\begin{aligned}
[1-\lambda\eta(\mu_D+\omega)]\sum_{m=1}^{M-2}(L^{M-1}-L^m)\delta^m\lambda\eta(\mu_D+\omega)L^{M-1} &< [1-\lambda\eta(\mu_D+\omega)]\delta^M L^M \\
\lambda\eta(\mu_D+\omega)\left[\sum_{m=1}^{M-2}(L^{M-1}-L^m)\delta^m-\delta^M L^M-L^{M-1}\right] &> \sum_{m=1}^{M-2}(L^{M-1}-L^m)\delta^m-\delta^M L^M \\
\frac{\sum_{m=1}^{M-2}(L^{M-1}-L^m)\delta^m-\delta^M L^M}{\eta(\mu_D+\omega)[\sum_{m=1}^{M-2}(L^{M-1}-L^m)\delta^m-\delta^M L^M-L^{M-1}]} &> \lambda
\end{aligned}
$$

Then the condition above makes $X_{i1}^{1-\lambda,M-1}$ positive. ■

Proof of Proposition 4

Proof. If $\sum_{m=1}^{M-1}(L^M-L^m)\delta^m-L^M\geq 0$, then $\frac{\sum_{m=1}^{M-1}(L^M-L^m)\delta^m-\eta(\mu_D+\omega)L^M}{\sum_{m=1}^{M-1}(L^M-L^m)\delta^m-L^M}>1$, which produce a contradiction with $\lambda\eta(\mu_D+\omega)<1$. By the contradiction it can conclude that $\sum_{m=1}^{M-1}(L^M-L^m)\delta^m-L^M<0$. Then

$$
\begin{aligned}
\sum_{m=1}^{M-1}(L^M-L^m)\delta^m+\lambda\eta(\mu_D+\omega)L^M &\geq \eta(\mu_D+\omega)\left[\lambda\sum_{m=1}^{M-1}(L^M-L^m)\delta^m+L^M\right] \\
\lambda\eta(\mu_D+\omega)\left[\sum_{m=1}^{M-1}(L^M-L^m)\delta^m-L^M\right] &\leq \sum_{m=1}^{M-1}(L^M-L^m)\delta^m-\eta(\mu_D+\omega)L^M \\
\lambda &\geq \frac{\sum_{m=1}^{M-1}(L^M-L^m)\delta^m-\eta(\mu_D+\omega)L^M}{\eta(\mu_D+\omega)[\sum_{m=1}^{M-1}(L^M-L^m)\delta^m-L^M]} \\
&\Longrightarrow X_{i1}^{\lambda,M}\leq 0.
\end{aligned}
$$

■
