# Supplementary material for: Simulation design to find the welfare impacts of livestock trading and disease transmission
Source: PLoS One. 2024 Nov 20;19(11):e0310213. doi: 10.1371/journal.pone.0310213 (PMC11578522; doi:10.1371/journal.pone.0310213)
Supplement: Attachment — Submitted filename: Response to Reviewers.pdf [file pone.0310213.s002.pdf]

Response to Reviewers

I am grateful for the helpful comments. To ease referencing to the comments, the reviewr comments are repeated in *italics* below, and my responses are in plain font.

Response to the Editor

Editor Comment — Thank you for stating the following financial disclosure: [Kyungpook National University Research Fund, 2022]. Please state what role the funders took in the study. If the funders had no role, please state: "The funders had no role in study design, data collection and analysis, decision to publish, or preparation of the manuscript."If this statement is not correct you must amend it as needed.Please include this amended Role of Funder statement in your cover letter; we will change the online submission form on your behalf.

Reply: The funders had no role in study design, data collection and analysis, decision to publish, or preparation of the manuscript.

Editor Comment — Thank you for stating the following in the Acknowledgments Section of your manuscript: [This paper is based on the fourth chapter of my Ph.D. dissertation. [This research was supported by Kyungpook National University Research Fund, 2022.] We note that you have provided funding information that is not currently declared in your Funding Statement. However, funding information should not appear in the Acknowledgments section or other areas of your manuscript. We will only publish funding information present in the Funding Statement section of the online submission form. Please remove any funding-related text from the manuscript and let us know how you would like to update your Funding Statement. Currently, your Funding Statement reads as follows:

Reply: I removed the following statement in the Acknowledgements Section: [This research was supported by Kyungpook National University Research Fund, 2022.]

Editor Comment — Thank you for updating your data availability statement. You note that your data are available within the Supporting Information files, but no such files have been included with your submission. At this time we ask that you please upload your minimal data set as a Supporting Information file, or to a public repository such as Figshare or Dryad. Please also ensure that when you upload your file you include separate captions for your supplementary files at the end of your manuscript.

Reply: This study is a simulation-based study, and the data used in the simulation are summarized in Tables 2 and 3 of the manuscript. Therefore, since no data was actually acquired and used externally, there is no data included in the requested Supporting Information File. For the statement, [All relevant data are within the manuscript and its Supporting Information files.], please understand that the statement means that all information is included in the manuscript.

Editor Comment — We note that your manuscript is not formatted using one of PLOS ONE's accepted file types. Please reattach your manuscript as one of the following file types: .doc, .docx,

.rtf, or .tex (accompanied by a .pdf). If your submission was prepared in LaTex, please submit your manuscript file in PDF format and attach your .tex file as "other."

Reply: I submitted the manuscript file in PDF format and attached .tex file as other.

Editor Comment — We note that several of your files are duplicated on your submission. Please remove any unnecessary or old files from your revision, and make sure that only those relevant to the current version of the manuscript are included.

Reply: I checked the duplicated files (manuscripts). I removed the unnecessary files from my revision.

Reviewer 1

Major comments:

Reviewer 1.1 — *In formula (2) of the article, it is mentioned that the goal of Agent is to maximize* π_{it}*, but in formula (4), it is mentioned again that the goal of Agent is to maximize* $U(C_{it})$*. Please check the wording of the article.*

Reply: Thank you for your thorough comments. What you mentioned is the theoretical aspect of the household production model. To explain this in detail in the paper, lines 141-143 are expanded as follows. "In the household production model households are both consumers and suppliers themselves, so both profit maximization and utility maximization are considered."

Reviewer 1.2 — *When drawing, all results can be drawn instead of just showing the total number of livestock.*

Reply: Your comment is very helpful. However, the currently presented graphical results are calculated through Monte-Carlo iteration, making it realistically difficult to present all results. And since the remaining results are also determined by cumulative values, there is not much information other than the current results. Therefore, it is judged that it is better to keep the results as the current figures. This is a good idea, but I would appreciate it if you could understand that it is difficult to implement in reality.

Reviewer 1.3 — *The maximum number of livestock populations set in the article is 5, and more types can be further discussed.*

Reply: Thank you for your constructive comments. The livestock population of 5 you mentioned is just one value used in the simulation, and larger numbers of M-types need to be addressed. Since one of the main limitations of this study is the parameterazation of the simulation model, this was given more emphasis in the discussion section to explicitly indicate this (line 930 - 943).

Reviewer 2

Major comments:

Reviewer 2.1 — *The abstract needs to be summarized and condensed, as it currently contains redundant information.*

Reply: I changed the abstract following your recommendations. The redundant parts of the abstract were removed and rewritten to be more dense.

Reviewer 2.2 — *The symbol representation in this paper is highly confusing, with issues of misused, disorderly, and reused key variables.*

Reply: First of all, I apologize for any confusion during the paper review process due to the notations in the paper. I have re-examined the parts you pointed out and re-checked and corrected the overall notation, including errors pointed out in other comments.

Reviewer 2.3 — *The actions and states of agents may be too simplistic; additional actions and states could be considered. It might be beneficial to define the agent states (e.g., livestock holding, liquid assets) and actions (e.g., buy/sell/do not trade), agent strategies (e.g., consumption-smoothing strategy), and agent types (uninformed, selfish, altruistic) before introducing the model.*

Reply: The limitations you pointed out are very important. I also deeply sympathize with the issue. It is realistically difficult to completely solve the problem in the current study. To specify the problem you, I mentioned it as a limitation on lines 943-946 of the Discussion section.

Reviewer 2.4 — *Is there a theoretical basis or related analysis for the production function in Equation 1?*

Reply: The theoretical background of Equation 1 is often covered in agricultural economics, especially economic theory related to farms. To specify this part, a new reference was added at number [26] (Kay, R., Edwards, W. & Duffy, P. Farm Management. (McGraw-Hill Education, 2019)) and the reference was revealed at line 157. Also, I changed the term "production function" as "single input production fuction" in 156 line.

Reviewer 2.5 — *For the constraint in Equation 5, since* $(X_{i,t})$ *represents net purchase, the positivity or negativity of* $(X_{i,t})$ *should be considered. A more detailed explanation of* $(X_{i,t})$ *at its first mention or in Equation 5 is warranted.*

Reply: I added the following to lines 220-223 to explain the constraints on net purchase in Equation (3): "In other words, a positive net purchase means that a household is purchasing livestock, and it is impossible to purchase more than the assets of the entire household. If net purchase is negative, this means that livestock is being sold, and there are no restrictions based on assets."

Reviewer 2.6 — *In the simulation section, explanations of variable values should be added. Different parameter choices in ABM can greatly influence simulation results.*

Reply: As you mentioned, settings for parameters in simulation are critical to the model results. In this study, very brief simulation results were presented according to the theoretical model results. Therefore, the results may vary depending on how the values of the simulation variables are selected. Since the main contribution of this study is to provide a simulation design, I explicitly mentioned the problematic issues as limitations (line 933 - 946).

Reviewer 2.7 — *Using vector graphics for the figures in the paper could enhance readability.*

Reply: Thank you so much for your constructive comments. I tried to re-implement the results with vector graphics as you suggested, but I found that the results by type were not more visible than the currently presented figures, so using vector graphics was not currently appropriate. I do not know if I am confusing vector graph and vector graphics, but I think the intention is conveyed well through the current figures.

Minor comments:

Reviewer 2.8 — *There's a formatting error in the quotation marks in* $F(L_{it})$ *above Equation 1, and the notation for* (L_{it}) *here differs from its form in the equation.*

Reply: I fixed that error. The modification can be seen at line 156.

Reviewer 2.9 — *The phrase "and t" inside the parentheses on line 156 is unrelated to the context and should be removed.*

Reply: I fixed that error. The modification can be seen at line 156.

Reviewer 2.10 — *The (X) in line 178 should match the* (X_{it}) *in Equation 2.*

Reply: I fixed that error. The modification can be seen at line 180.

Reviewer 2.11 — *The meaning of* (C_{it}) *in Equation 3 is not introduced.*

Reply: I added information about C_{it} to explain the meaning in the Equation (3) in lines 189-190.

Reviewer 2.12 — *Some names need to be standardized; for example, "farmer" on line 200 and "farmers" on line 224 should be consistently referred to as "household" unless they have different meanings in the model, in which case they should be clearly defined and indicated in the subscripts of the symbols. Additionally, "product consumption, denoted as C" on line 205 differs from Equation 3 and should be specifically described, with the symbol's meaning made clearer.*

Reply: Thank you for your detailed comments. I left the mention of farm or farmers in the introduction for explanation of related research and changed the remaining notations to household according to your suggestion. These revisions were made to the entire paper, and the revisions are marked in purple. I also changed the typo in line 207 for C to C_{it}.

Reviewer 2.13 — *(C_t) on line 213 should be (C_{it}), and the same applies to line 215. The author needs to carefully check and correct the symbol system in the paper to ensure consistency in symbol meanings. Further instances of symbol misuse are not elaborated here.*

Reply: I fixed that error. The modification can be seen at line 215. And as I mentioned earlier, I checked the entire paper again for the notations you pointed out.

Reviewer 2.14 — *Both Equation 2 and Equation 5 (the equilibrium price on line 257) include (p_t), but they represent different meanings.*

Reply: The meaning of the equilibrium price is the same in both Equations (2) and (5). To make this clear, lines 265-266 reveal the simultaneity of price variable determination.

Reviewer 2.15 — *The meaning of the variable (α) in Equation 6 is not explained.*

Reply: I added the explanation about α in lines 270-271.
